# Histology-dependent prognostic role of pERK and p53 protein levels in early-stage non-small cell lung cancer

**DOI:** 10.18632/oncotarget.24977

**Published:** 2018-04-13

**Authors:** Álvaro Quintanal-Villalonga, Mariló Mediano, Irene Ferrer, Ricardo Meléndez, Andrés Carranza-Carranza, Rocío Suárez, Amancio Carnero, Sonia Molina-Pinelo, Luis Paz-Ares

**Affiliations:** ^1^ H120-CNIO Lung Cancer Clinical Research Unit, Instituto de Investigación 12 de Octubre and CNIO, Madrid, Spain; ^2^ Instituto de Biomedicina de Sevilla (IBIS) (HUVR, CSIC, Universidad de Sevilla), Sevilla, Spain; ^3^ Hospital Universitario Virgen del Rocío (HUVR), Sevilla, Spain; ^4^ Medical Oncology Department, Hospital Universitario Doce de Octubre & Centro Nacional de Investigaciones Oncológicas (CNIO), Madrid, Spain; ^5^ Medical School, Universidad Complutense, Madrid, Spain; ^6^ CiberOnc, Madrid, Spain

**Keywords:** p53, pERK, prognostic, biomarkers, NSCLC

## Abstract

Lung tumors represent a major health problem. In early stage NSCLC tumors, surgical resection is the preferred treatment, but 30-55% of patients will relapse within 5 years after surgery. Thus, the identification of prognostic biomarkers in early stage NSCLC patients, especially those which are therapeutically addressable, is crucial to enhance survival of these patients. We determined the immunohistochemistry expression of key proteins involved in tumorigenesis and oncogenic signaling, p53, EGFR, pAKT and pERK, and correlated their expression level to clinicopathological characteristics and patient outcome. We found EGFR expression is higher in the squamous cell carcinomas than in adenocarcinomas (p=0.043), and that nuclear p53 staining correlated with lower differentiated squamous tumors (p=0.034). Regarding the prognostic potential of the expression of these proteins, high pERK levels proved to be an independent prognostic factor for overall (p<0.001) and progression-free survival (p<0.001) in adenocarcinoma patients, but not in those from the squamous histology, and high p53 nuclear levels were identified as independent prognostic factor for progression-free survival (p=0.031) only in squamous cell carcinoma patients. We propose a role as early prognostic biomarkers for pERK protein levels in adenocarcinoma, and for nuclear p53 levels in squamous cell lung carcinoma. The determination of these potential biomarkers in the adequate histologic context may predict the outcome of early stage NSCLC patients, and may offer a therapeutic opportunity to enhance survival of these patients.

## INTRODUCTION

Lung tumors represent a major health problem, accounting for most cancer-related deaths and with a 5-year survival rate of only 18% after diagnosis [1]. Lung cancer is a heterogeneous disease and is classified into two major groups: small-cell lung cancer (SCLC) and non-small cell lung cancer (NSCLC) [2]. NSCLC is the most common histology of lung cancer, representing 85% of lung cancer cases and is sub-classified as adenocarcinoma, squamous cell lung cancer and large cell carcinoma. Surgical resection plays a major role in the therapy of early-stage NSCLC tumors. After resection, recurrence, which occurs in up to 30-55% of patients at 5 years post-surgery, and the appearance of distant metastases will determine the outcome [3–5]. Thus, the identification of prognostic biomarkers in early-stage NSCLC patients, especially those which are therapeutically addressable, is crucial to enhance survival of these patients.

There is evidence that different molecular alterations underlie phenotypic differences in NSCLC. These alterations are clinically relevant, and some of them represent feasible targets, with therapeutic implications [6]. In this sense, lung cancer patients harboring EGFR mutations are sensitive to EGFR tyrosine kinase inhibitors (TKIs) [7]. Although these mutations are characteristic of adenocarcinoma tumors [8, 9], it has been recently shown that some squamous cell carcinoma patients respond to anti-EGFR therapy [10] and that high EGFR expression levels correlate with better responses in these patients [11], highlighting the therapeutic relevance of EGFR in this setting. Alterations in EGFR, as well as in other genes such as KRAS mutations or ALK translocations, are frequent in lung adenocarcinoma; all these changes have been involved in the activation of signaling pathways critical in lung tumorigenesis, such as MAPK and PI3K/AKT pathways [12–14]. In the MAPK pathway, the phosphorylation of p42/p44 (ERK) is central, leading to their translocation to the nucleus, where they act as transcription factors and activate the expression of genes related to cell proliferation, anti-apoptosis, differentiation, migration and angiogenesis [15, 16]. In the PI3K/AKT pathway, the most important effector is AKT, whose phosphorylation leads to the activation of downstream proteins, which trigger pro-survival signaling, inhibit several repressors of cell cycle, and induce the transcription of pro-angiogenic genes [17].

On the other hand, the role of p53 is crucial in tumor suppression. When active, this protein binds to DNA and induces the expression of genes leading to cell cycle arrest and apoptosis. However, mutant p53 is unable to bind DNA and can no longer prevent cell cycle continuation, which contributes to cancer progression [18]. Furthermore, mutant p53 cannot be efficiently degraded and accumulates in the nucleus; thus, its protein levels can be easily detected [19]. To elucidate the role of these pathways in the tumorigenesis of NSCLC, we have determined the protein expression levels of key players in NSCLC, including EGFR, pAKT, pERK, and p53, as prognostic biomarkers in early-stage NSCLC.

## RESULTS

### Correlation of pAKT, pERK, nuclear p53 and EGFR protein levels and clinicopathological features

This study involved a cohort of 248 NSCLC patients with early-stage NSCLC, who were surgically resected (Table 1). Most patients were men (94.0%) with a median age of 66 years [interquartile range 39-84], with a generally good performance status (ECOG 0-1 in 96.3% patients). Most of them were current or ex-smokers (48.4% and 45.6%, respectively), while only 4.4% were never-smokers. Considering the histology, 49.6% and 31.0% cases belonged to the most prevalent lung cancer subtypes, squamous cell lung carcinoma and adenocarcinoma, respectively; 8.9% cases were diagnosed as large cell carcinomas; and 10.4% were identified as other histologic subtypes. Most tumors showed modest differentiation, with 39.4% and 31.5% of tumors classified as poorly or moderately differentiated, respectively, while only 7.3% of tumors showed high differentiation levels. Most tumors were diagnosed at stage I (56.9%) or II (27.8%), and only 14.9% cases were diagnosed at stage IIIA. Following radical surgery, 12.1% patients received adjuvant radiotherapy, and 9.7% patients received adjuvant chemotherapy. Adjuvant chemotherapy was offered to patients with resected stage II and III NSCLC and in some patients with resected stage IB disease and a primary tumor larger than 4 cm. During the time of patient monitoring, 48.0% patients relapsed and 61.7% died, the most frequent cause for death being tumor progression. Baseline characteristics of the patients with adenocarcinoma (N=77) and squamous cell carcinoma (N=124) from this cohort are shown in Table 2. The clinicopathological characteristics of both adenocarcinoma and squamous cell carcinoma cohorts were similar. Differences between the two patient subsets were observed only in gender distribution (p=0.008); in the smoking status of patients, with a higher percentage of current smokers and a lower percentage of never-smokers in the squamous cell carcinoma cohort (p=0.024); and in tumor differentiation levels, with a higher percentage of poorly differentiated tumors in the squamous cell carcinoma cohort (p<0.001).

**Table 1:** Clinicopathological characteristics of the NSCLC cohort

Variable	NSCLC (N=248)
**Gender**	
Male	233(94.0%)
Female	15(6.0%)
**ECOG Performance status**	
0	167(67.3%)
1	72(29.0%)
2	3(1.2%)
**Age**	66 [39–84]
**Smoking habits**	
Ex smoker	113(45.6%)
Current smoker	120(48.4%)
Never smoker	11 (4.4%)
**Histology**	
Squamous cell carcinoma	123 (49.6%)
Adenocarcinoma	77 (31.0%)
Large cell carcinoma	22 (8.9%)
Other	26 (10.4%)
**Stage**	
IA	27 (10.9%)
IB	114 (46.0%)
IIA	4 (1.6%)
IIB	65 (26.2%)
IIIA	37 (14.9%)
**Tumour differentiation**	
Well differentiated	18 (7.3%)
Moderately differentiated	78 (31.5%)
Poorly differentiated	98 (39.4%)
**Adjuvant radiotherapy**	
Yes	30 (12.1%)
No	205 (82.7%)
**Adjuvant chemotherapy**	
Yes	24 (9.7%)
No	213 (85.9%)
**Relapse**	
Yes	119 (48.0%)
No	109 (44.0%)
**Exitus**	
Yes	153 (61.7%)
No	76 (30.6%)
**Reason for Exitus**	
Progression	104 (41.9%)
Not related	22 (8.9%)
Undetermined	107 (43.1%)
Surgical complications	4 (1.6%)
Toxicity	10 (4.0%)
Second tumour	1 (0.4%)

Continuous variables are expressed as median [interquartile range] and categorical variables are expressed as the number of cases (percentage).

**Table 2:** Clinicopathological characteristics of the ADC and the SCC patient subsets in the NSCLC cohort

Variable	ADC (N=77)	SCC (N=124)	p-value
**Gender**			
Male	69 (89.6%)	122 (98.4%)	
Female	8 (10.4%)	2 (1.6%)	p=0.008
**ECOG Performance status**			
0	50 (64.9%)	82 (66.1%)	
1	24 (31.2%)	36 (29.0%)	
2	1 (1.3%)	2 (1.6%)	p=0.945
**Age**	63 [40–81]	67 [41–84]	p=0.857
**Smoking habits**			
Ex smoker	35 (45.5%)	56 (45.2%)	
Current smoker	34 (44.2%)	66 (53.2%)	
Never smoker	6 (7.8%)	1 (0.8%)	**p=0.024**
**Stage**			
IA	5 (6.5%)	20 (16.1%)	
IB	42 (54.4%)	48 (38.7%)	
IIA	1 (1.3%)	3 (2.4%)	
IIB	18 (23.4%)	32 (25.8%)	
IIIA	10 (13.0%)	21 (16.9%)	p=0.130
**Tumour differentiation**			
Well differentiated	11 (14.3%)	5 (4.9%)	
Moderately differentiated	26 (33.8%)	49 (39.5%)	
Poorly differentiated	24 (31.2%)	63 (50.8%)	**p<0.001**
**Adjuvant radiotherapy**			
Yes	11 (14.3%)	14 (11.3%)	
No	63 (81.8%)	101 (81.5%)	p=0.373
**Adjuvant chemotherapy**			
Yes	10 (13.7%)	7 (6.0%)	
No	63 (57.1%)	110 (94.0%)	p=0.062
**Relapse**			
Yes	40 (51.9%)	59 (47.6%)	
No	33 (42.9%)	53 (42.7%)	p=0.448
**Exitus**			
Yes	48 (62.3%)	80 (64.5%)	
No	26 (33.8%)	32 (25.8%)	p=0.216

Continuous variables are expressed as median [interquartile range] and categorical variables are expressed as the number of cases (percentage).

**Figure 1:**
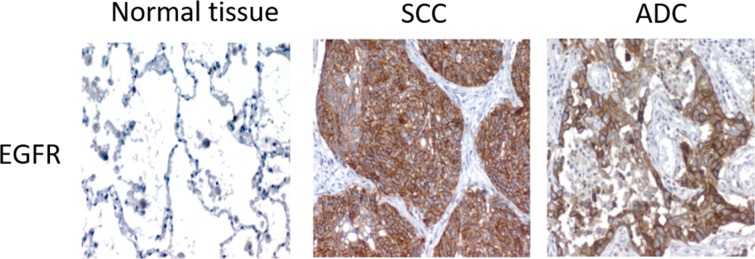
Representative images of the different IHC detection patterns of EGFR in squamous cell carcinoma and adenocarcinoma patients

We determined the protein levels of pAKT, pERK, nuclear p53 and EGFR by IHC in the tumor samples of the entire stage I-IIIA NSCLC cohort and related them to the clinical information available. EGFR staining was positive in 69.9% of samples, and higher levels of this protein were associated with the squamous tumor histology (p=0.043, odds ratio of 2.222, 95% CI 1.207-3.993, Table 3, Figure 1). pAKT staining was detected in 68.7% of samples, but no association was observed between this result and clinical features. When the squamous-cell-carcinoma-patient cohort was independently analyzed (Table 4), a correlation was observed between pERK and smoking habits, with a high pERK IHC score observed in a lower percentage of current smokers (p=0.030, odds ratio of 0.425, 95% CI 0.188-0.959), compared with the remaining squamous cell carcinoma patients (Table 4). Nuclear p53 staining was detected in 97.2% of samples, and a correlation was observed between higher percentage of nuclear p53 staining and lower tumor differentiation when the entire cohort was analyzed (p=0.01, odds ratio of 2.002, 95% CI 1.207-3.323, Table 3). However, when we analyzed the adenocarcinoma- and squamous-cell-carcinoma-patient subsets independently, this correlation was only detected in the squamous cell carcinoma tumors (p=0.034, odds ratio of 2.109, 95% CI 1.011-4.400, Table 4) but not in the adenocarcinoma samples under study (p=0.188, Table 5). In addition, a correlation was observed between age and nuclear p53 levels within adenocarcinoma patients, with older patients showing a decreased percentage of nuclear-p53-positive staining (p=0.018, odds ratio of 0.356, 95% CI 0.135-0.938, Table 5).

**Table 3:** Association between protein levels of pAKT, pERK, nuclear p53, and EGFR and clinicopathological characteristics of the NSCLC cohort (N=248)

	EGFR expression level		pAKT nuclear expression level		pERK nuclear expression level		p53 nuclear %	
	**0-1**	**2-3**	**0-1**	**2-3**	**0-1**	**2-3**	**<10%**	**≥10%**
Gender^**^								
**Male**	109 (94.8)	123(93.2)	105 (94.6)	122 (93.1)	**157 (91.8)**	**70 (98.6)**	114 (92.7)	130 (94.9)
**Female**	6 (5.2)	9 (6.8)	6 (5.4)	9 (6.9)	**14 (8.2)**	**1 (1.4)**	9(7.3)	7(5.1)
**p-value**		p=0.401		p=0.422		**p=0.035**		NS
Age^**^								
**<66 yo**	53 (46.1)	66 (50.0)	58 (52.3)	59 (45.0)	85 (49.7)	32(45.1)	60(48.8)	67(48.9)
**≥66 yo**	62 (53.9)	66 (50.0)	53 (47.7)	72 (55.0)	86 (50.3)	39(54.9)	63(51.2)	70(51.1)
**p-value**		p=0.313		p=0.161		p=0.303		NS
ECOG^**^								
**0**	81 (72.3)	86 (66.7)	71 (67.0)	93 (71.5)	118(71.1)	46(65.7)	83(68.6)	88(66.2)
**Rest**	31 (27.7)	43 (33.3)	35 (33.0)	37 (28.5)	48 (28.9)	24(34.3)	38(31.4)	45(33.8)
**p-value**		p=0.209		p=0.269		p=0.252		NS
Smoking habits^**^								
**Current smoker**	50 (43.5)	69 (52.3)	54 (48.6)	63 (48.1)	86 (50.3)	31(43.7)	62(50.4)	63(46.0)
**Rest**	65 (56.5)	63 (47.7)	57 (51.4)	68 (51.9)	85 (49.7)	40(56.3)	61(49.6)	74(54.0)
**p-value**		p=0.105		p=0.517		p=0.212		NS
Histology^**^								
**SCC**	**41 (50.6)**	**82 (69.5)**	53 (58.2)	67 (63.8)	86 (63.2)	34 (58.6)	56(50.5)	73(59.8)
**ADC**	**40 (49.4)**	**36 (30.5)**	38 (41.8)	38 (36.2)	52 (37.7)	24(41.4)	55(49.5)	49(40.2)
**p-value**		**p=0.043**		p=0.257		p=0.371		NS
Tumor differentiation^**^								
**Poor**	48 (41.7)	49 (37.1)	38 (34.2)	58 (44.3)	63 (36.8)	33(46.5)	**39(31.7)**	**66(48.2)**
**Rest**	67 (58.3)	83 (62.9)	73 (65.8)	73 (55.7)	108 (63.2)	38(53.5)	**84(68.3)**	**71(51.8)**
**p-value**		p=0.271		p=0.072		p=0.106		**p=0.01**
Stage^**^								
**I**	67 (58.3)	73 (55.3)	59 (53.2)	77 (58.8)	92 (53.8)	44(62.0)	66(53.7)	71(51.8)
**II/III**	48 (41.7)	59 (44.7)	53 (46.8)	54 (41.2)	79 (46.2)	27(38.0)	57(46.3)	66(48.2)
**p-value**		p=0.367		p=0.227		p=0.153		NS

Categorical values are expressed as number of cases (percentage)

^*^p-values were obtained using the Chi-square test. p-values are considered significant when lower than 0.05.

^**^ IHC staining about some cases on these variables was not available.

**Table 4:** Association between protein levels of pAKT, pERK, nuclear p53, and EGFR and clinicopathological characteristics of the SCC cohort (N=124)

	EGFR expression level		pAKT nuclear expression level		pERK nuclear expression level		p53 nuclear %	
	0-1	2-3	0-1	2-3	0-1	2-3	<10%	≥10%
Gender^**^								
**Male**	48 (98.0)	73 (98.6)	53	65 (97.0)	85 (98.8)	33(97.1)	51 (98.1)	67 (98.5)
**Female**	1 (2.0)	1 (1.4)	(100.0)	2 (3.0)	1 (1.2)	1 (2.9)	1 (1.9)	1 (1.5)
**p-value**		p=0.640	0 (0.0)	p=0.310		p=0.488		p=0.681
Age^**^								
**<66 yo**	19 (38.8)	36 (48.6)	24 (45.3)	30 (44.8)	43 (50.0)	11(32.4)	27 (51.9)	27 (39.7)
**≥66 yo**	30 (61.2)	38 (51.4)	29 (54.7)	37 (55.2)	45 (50.0)	23 67.6)	25 (48.1)	41 (60.3)
**p-value**		p=0.186		p=0.551		p=0.060		p=0.126
ECOG^**^								
**0**	34 (72.3)	48 (66.7)	30 (60.0)	50 (75.8)	0 (72.3)	20 60.6)	35 (70.0)	45 (68.2)
**Rest**	13 (27.7)	24 (33.3)	20 (40.0)	16 (24.2)	23 (27.7)	13 (39.4)	15 (30.0)	21 (31.8)
**p-value**		p=0.328		p=0.054		p=0.157		p=0.499
Smoking habits^**^								
**Current smoker**	23 (46.9)	42 (56.8)	27 (50.9)	37 (55.2)	**51 (59.3)**	**13 38.2)**	29 (55.8)	35 (51.5)
**Rest**	26 (53.1)	32 (43.2)	26 (49.1)	30 (44.8)	**35 (40.7)**	**21 61.8)**	23 (44.2)	33 (48.5)
**p-value**		p=0.189		p=0.389		**p=0.030**		p=0.389
Tumor differentiation^**^								
**Poor**	27 (55.1)	35 (47.3)	22 (41.5)	39 (58.2)	41 (47.7)	20(58.8)	**21 (40.4)**	**40 (58.8)**
**Rest**	22 (44.9)	39 (52.7)	31 (58.5)	28 (41.8)	45 (52.3)	14(41.2)	**31 (59.6)**	**28 (41.2)**
**p-value**		p=0.254		p=0.051		p=0.185		**p=0.034**
Stage^**^								
**I**	27 (55.1)	41 (55.4)	28 (52.8)	38 (56.7)	45 (52.3)	21(61.8)	28 (53.8)	38 (55.9)
**II/III**	22 (44.9)	33 (44.6)	25 (47.2)	29 (43.3)	41 (47.7)	13(38.2)	24 (46-2)	30 (44.1)
**p-value**		p=0.560		p=0.405		p=0.232		p=0.485

Categorical values are expressed as number of cases (percentage)

^*^p-values were obtained using the Chi-square test. p-values are considered significant when lower than 0.05.

^**^ IHC staining about some cases on these variables was not available.

**Table 5:** Association between protein levels of pAKT, pERK, nuclear p53, and EGFR and clinicopathological characteristics of the ADC cohort (N=77)

	EGFR expression level		pAKT nuclear expression level		pERK nuclear expression level		p53 nuclear %	
	0-1	2-3	0-1	2-3	0-1	2-3	0-1	2-3
Gender^**^								
**Male**	36 (90.0)	32(88.9)	35 (92.1)	33 (86.8)	44	24(100.0)	34(85.0)	33(94.3)
**Female**	4 (10.0)	4 (11.1)	3 (7.9)	5 (13.2)	(84.6)	0 (0.0)	6 (15.0)	2 (5.7)
**p-value**		p=0.583		p=0.356	8 (15.4)	p=0.07		p=0.179
Age^**^								
**<66 yo**	25 (62.5)	19(52.8)	24 (63.2)	20 (52.6)	30(57.7)	14 (58.3)	**18(45.0)**	**25(71.4)**
**≥66 yo**	15 (37.5)	17(47.2)	14 (36.8)	18 (47.4)	22(42.3)	10 (41.7)	**22(55.0)**	**10(28.6)**
**p-value**		p=0.266		p=0.243		p=0.580		**p=0.018**
ECOG^**^								
**0**	28 (71.8)	22(62.9)	26 (72.2)	24 (63.2)	36(72.0)	14 (58.3)	28(70.0)	21(63.6)
**Rest**	11 (28.2)	13(37.1)	10 (27.8)	14 (36.8)	14(28.0)	10 (41.7)	12(30.0)	12(36.4)
**p-value**		p=0.284		p=0.280		p=0.181		p=0.372
Smoking habits^**^								
**Current smoker**	16 (40.0)	17(47.2)	19 (50.0)	14 (36.8)	21(40.4)	12 (50.0)	18(45.0)	14(40.0)
**Rest**	24 (60.0)	19(52.8)	19 (50.0)	24 (63.2)	31(59.6)	12 (50.0)	22(55.0)	21(60.0)
**p-value**		p=0.344		p=0.177		p=0.295		p=0.420
Tumor differentiation^**^								
**Poor**	14 (35.0)	9 (25.0)	10 (26.3)	13 (34.2)	15(28.8)	8 (33.3)	10(25.0)	13(37.1)
**Rest**	26 (65.0)	27(75.0)	28 (73.7)	25 (65.8)	37(71.2)	16 (66.7)	30(75.0)	22(62.9)
**p-value**		p=0.243		p=0.309		p=0.444		p=0.188
Stage^**^								
**I**	25 (62.5)	21(58.3)	20 (52.6)	26 (68.4)	32(61.5)	14 (58.3)	24(60.0)	22(62.9)
**II/III**	15 (37.5)	15(41.7)	18 (47.4)	12 (31.6)	20(38.5)	10 (41.7)	16(40.0)	13(37.1)
**p-value**		p=0.446		p=0.120		p=0.492		p=0.494

Categorical values are expressed as number of cases (percentage)

^*^p-values were obtained using the Chi-square test. p-values are considered significant when lower than 0.05.

^**^ IHC staining about some cases on these variables was not available.

### Role of pAKT, pERK, nuclear p53 and EGFR protein levels as prognostic biomarkers in early-stage NSCLC

**Figure 2:**
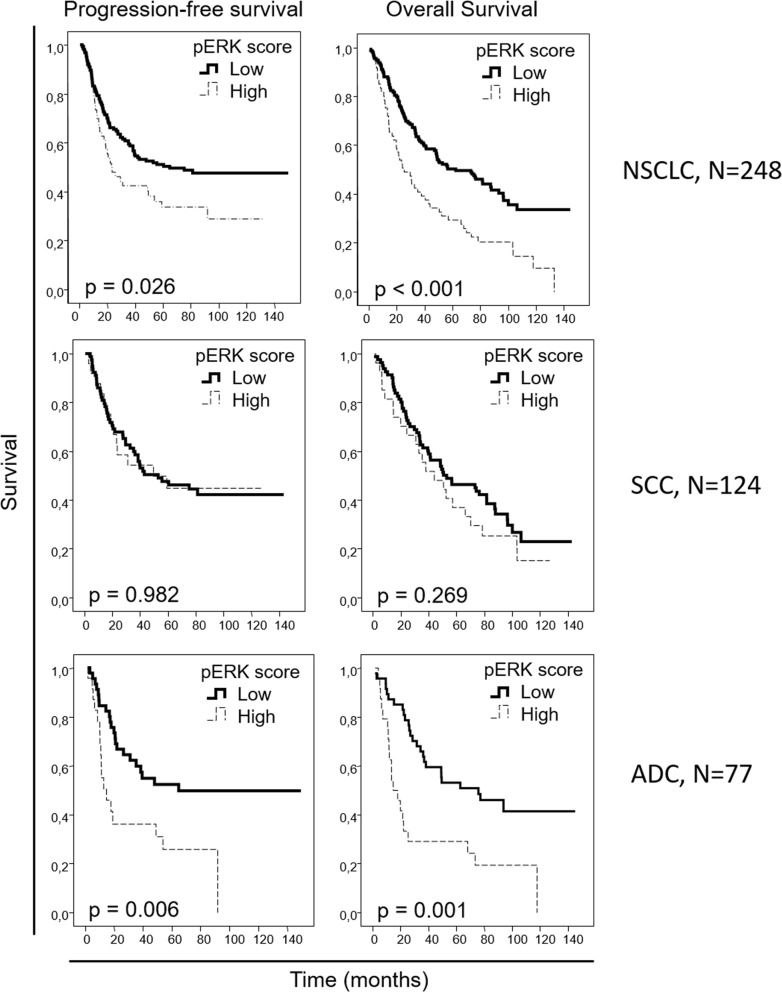
Overall and progression-free Kaplan-Meier survival curves for the entire NSCLC patient cohort and for the squamous cell carcinoma (SCC) and adenocarcinoma (ADC) patients, attending to the pERK score, as assessed by IHC Scores of 0 and 1 were considered as “low”, and scores of 2 or 3 were considered as “high”.

**Figure 3:**
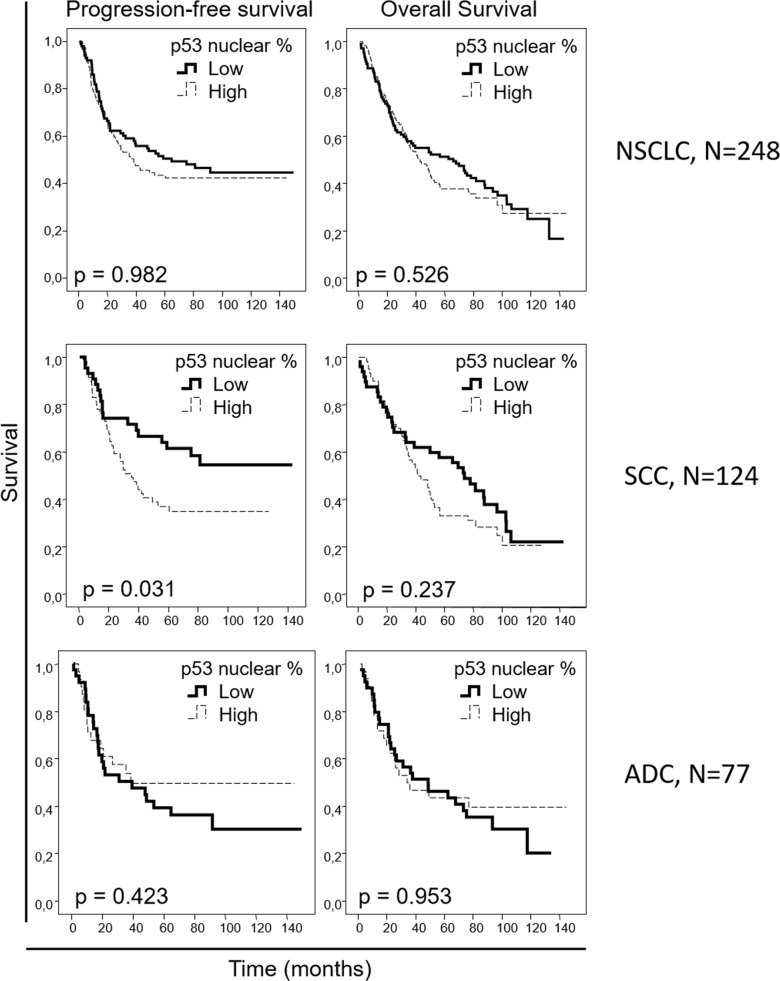
Overall and progression-free Kaplan-Meier survival curves for the entire NSCLC patient cohort and for the squamous cell carcinoma (SCC) and adenocarcinoma (ADC) patients, attending to the nuclear p53 percentage, as assessed by IHC Percentages less than 10% were considered as “low”, and percentages equal to or above 10% were considered as “high”.

We aimed to evaluate if the protein levels of our candidate biomarkers could be potential prognostic markers in early NSCLC; therefore, we correlated our IHC data with the clinical outcome information. When the entire NSCLC cohort was analyzed, no association was observed between nuclear pAKT or EGFR levels and survival in the univariate analysis (Supplementary Figures 1-2). However, higher pERK levels correlated with worse progression-free (p=0.026) and overall (p<0.001) survival, with median progression-free survivals of 20.3 [11.^8^–^28^.^8^] versus 54.9 [34.6-70.0] months and median overall survivals of 30.5 [16.3-44.6] versus 56.1 [35.4-76.8] months for patients with high- and low-pERK-staining tumors, respectively, when the entire NSCLC cohort was considered. When the squamous cell carcinoma patient cohorts were independently analyzed, no association of pERK levels with patient outcome was observed. However, when only the adenocarcinoma patient cohort was studied, a clear correlation was reported between higher pERK levels and worse progression-free (p=0.006) and overall (p=0.001) survival, with median progression-free survival not reached in the low-pERK-level group vs 14.5 [4.4-24.6] months in the high-pERK-level group and a median overall survival of 62.6 [21.9-103.3] versus 17.7 [7.5-28.0] months, respectively (Figure 2). When we analyzed the association between patient survival and nuclear p53 percentage from the IHC data (Figure 3), no association was found in the entire NSCLC cohort nor in the adenocarcinoma-patient subset. However, in the squamous cell carcinoma patients, a clear association was found between higher nuclear p53 percentage and worse progression-free survival (p=0.031), with a median progression-free survival of 35.4 [22.7-48.1] months for patients with high nuclear p53 staining, while the median survival value was not reached in the group of patients with low nuclear p53 protein levels.

In multivariate analysis performed in the entire cohort (Table 6), stage and ECOG performance status were found to be independent prognostic factors in progression-free (p=0.001 and p=0.010, respectively) and overall (p<0.001 and p=0.001, respectively) survival, as expected, as well as pERK levels (p=0.010 for progression-free and p<0.001 for overall survival). In the adenocarcinoma patient subset, the stage was reported as an independent prognostic factor, correlating with poorer progression-free (p=0.006) and overall (p=0.017) survival. Patient smoking habits and administration of adjuvant radiotherapy were identified as well to have an independent negative prognostic role in adenocarcinoma patients (Table 6), with respect to progression-free (p=0.001 and p=0.004, respectively) and overall (p=0.036 and p=0.006, respectively) survival, as well as pERK levels (p<0.001 and p<0.001, respectively). In multivariate analysis of the squamous-cell-carcinoma-patient subset (Table 6), ECOG demonstrated an independent prognostic role in progression-free (p=0.003) and overall (p=0.001) survival. Nuclear p53 protein levels proved to be an independent prognostic factor in the squamous histology patient cohort, with respect to progression-free survival (p=0.031).

**Table 6:** Multivariate analysis of variables independently affecting outcome in the whole cohort (N=248)

	NSCLC (N=248)				Adenocarcinoma (N=77)				Squamous cell carcinoma (N=124)			
	PFS		OS		PFS		OS		PFS		OS	
Variable	Hazard ratio (IC 95%)	p-value	Hazard ratio (IC 95%)	p-value	Hazard ratio (IC 95%)	p-value	Hazard ratio (IC 95%)	p-value	Hazard ratio (IC 95%)	p-value	Hazard ratio (IC 95%)	p-value
**Nuclear pAKT score**	0,91 (0.61-1.34)	0,621	0,94 (0.66-1.32)	0,711	0,56 (0.27-1.17)	0,121	0,49 (0.25-1.10)	0,570	0,67 (0.37-1.22)	0,192	0,79 (0.48-1.31)	0,360
**Nuclear pERK score**	**1,70 (1.11-2.60)**	**0,010**	**2,06 (1.41-3.00)**	**<0,001**	**3,87 (1.73-8.63)**	**<0,001**	**4,30 (1.99-9.26)**	**<0,001**	0,80 (0.39-1.65)	0,548	1,12 (0.61-2-05)	0,710
**EGFR score**	1,12 (0.76-1.65)	0,563	1,11 (0.79-1.57)	0,545	1,19 (0.62-2.28)	0,619	0,94 (0.50-1.76)	0,855	1,28 (0.69-2.39)	0,432	1,30 (0.77-2.19)	0,322
**p53 nuclear %**	1,05 (0.72-1.55)	0,796	1,04 (0.74-1.47)	0,802	0,71 (0.30-1.71)	0,453	0,89 (0.39-2.04)	0,781	**1,96 (1.08-3.56)**	**0,031**	1,26 (0.78-2.03)	0,358
**Smoking habits**	1,18 (0.80-1.74)	0,393	1,06 (0.75-1.49)	0,756	**3,31 (1.47-7.47)**	**0,001**	**1,817 (1.041-3.171)**	**0,036**	1,11 (0.60-2.08)	0,732	1,09 (0.63-1.89)	0,757
**Stage**	**1,91 (1.30-2.81)**	**0,001**	**1,82 (1.29-2.56)**	**<0,001**	**2,18 (1.01-4.69)**	**0,006**	**1,474 (1.071-2.029)**	**0,017**	1,41 (0.78-2.55)	0,256	1,41 (0.85-2.33)	0,19
**Tumour differentiation**	0,83 (0.55-1.26)	0,391	0,94 (0.65-1.37)	0,756	1,08 (0.55-2-14)	0,814	1,09 (0.57-2.11)	0,793	1,06 (0.55-2.02)	0,866	1,30 (0.76-2.24)	0,344
**ECOG**	**1,72 (1.14-2.58)**	**0,010**	**1,95 (1.35-2.82)**	**0,001**	1,11(0.45-2.70)	0,828	1,89 (0.83-4.30)	0,132	**2,35 (1.28-4.33)**	**0,003**	**2,37 (1.39-4.03)**	**0,001**
**QT**	0,95 (0.84-1.07)	0,381	0,94 (0.84-1.06)	0,323	1,11 (0.89-1.39)	0,377	0,97 (0.78-1.21)	0,796	0,97 (0.78-1.21)	0,799	0,95 (0.78-1.16)	0,638
**RT**	1,73 (1.00-3.02)	0,078	1,68 (1.00-2.81)	0,067	**6,53 (2.18-19.61)**	**0,004**	**4,41 (1.55-12.58)**	**0.006**	1,87 (0.76-4,57)	0,173	1.95 (0.89-4.28)	0.095
**Histology**	1,12(0.97-1.28)	0,118	1,05 (0.93-1.20)	0,428	-	-	-	-	-	-	-	-

PFS: Progression-Free Survival; OS: Overall Survival

In addition, we studied the association of the expression of these proteins with the response to adjuvant chemotherapy. To this aim, we performed subset analysis in stage II-III patients as previously described [20]. By incorporating the expression levels of the previously assessed proteins into chemotherapy treatment information, we identified a trend demonstrating that patients with high nuclear pAKT expression could benefit from adjuvant chemotherapy; however, this therapeutic approach may not be a good choice for patients whose tumors show low expression of this marker (Supplementary Figure 3A). A contrasting trend was observed in the case of pERK levels; patients whose tumors showed high expression of pERK exhibited shorter survival times in the adjuvant chemotherapy arm, while low pERK expression in tumors correlated with better outcomes after adjuvant chemotherapy administration (Supplementary Figure 3B). However, these results did not reach statistical significance. This finding may be due to the low number of patients under adjuvant chemotherapy treatment in our cohort (9.7%, Table 1), which additionally precluded the feasibility of performing these analyses separating by tumor histology. For the other two proteins under study (EGFR and p53), however, we did not find any association of their expression to chemotherapy response (Supplementary Figure 4A-4B).

## DISCUSSION

In this study, we report a prognostic role for pERK levels in lung adenocarcinoma, and we demonstrate that nuclear p53 expression is a potential prognostic biomarker in lung squamous cell carcinoma patients, where it is associated with poorer tumor differentiation.

Within our early-stage NSCLC cohort, we analyzed the differences between the adenocarcinoma- and the squamous-cell-carcinoma-patient subsets to prevent any influence from important variations between the subsets on our conclusions. We observed that gender distribution between the subsets was different, with almost all women located in the adenocarcinoma subset. However, this is possibly due to the low number of women in our cohort (15 women versus 233 men). Furthermore, we found differences in the smoking habits of the patients, with a higher proportion of former or current smokers in the squamous cell carcinoma subset, compared to the adenocarcinoma subset. This is consistent with the literature, as squamous cell carcinoma has been linked to heavy smoking [21, 22]. We observed that tumors in the squamous cell carcinoma cohort showed poorer differentiation than those from the adenocarcinoma subset, but this may be an intrinsic characteristic of squamous cell carcinoma, as reported in other cohorts [23, 24]. No differences in other important clinicopathological variables were observed.

When we analyzed the relationships between pAKT, pERK, p53 and EGFR levels and clinical data, we observed that the squamous cell carcinoma tumors showed higher EGFR protein levels. EGFR has been primarily linked to adenocarcinoma, where alterations in this gene represent a cancer-driving force, and anti-EGFR therapy is approved for patients with these alterations. However, anti-EGFR therapy has proven efficacy in patients with the squamous cell carcinoma histology as well [10, 11]. Our data suggest that, although EGFR molecular alterations are not as frequent in squamous cell carcinoma as in adenocarcinoma, high expression of this gene occurs in the squamous cell carcinoma and may be the reason for anti-EGFR therapy being efficacious in some squamous cell carcinoma patients [11].

Regarding the prognostic potential of our candidate biomarkers, we found a prognostic role for the assessment of pERK nuclear levels in our early-stage NSCLC cohort. pERK levels have been observed to correlate with advanced staging, lymph node involvement and tumor size in NSCLC-patient cohorts including tumors at all stages [25, 26]. In several retrospective studies involving NSCLC patients, high pERK levels were reported as a prognostic factor for overall survival [26, 27] and for recurrence-free survival [28]. In these studies, unlike in our study, early- and late-stage tumors, adenocarcinomas and squamous cell carcinomas were jointly analyzed. In line with these results, when we analyzed our entire cohort, a prognostic role for pERK was observed in these early-stage tumors; however, further analysis by us revealed that this effect is exclusive for adenocarcinoma and that the prognostic role of pERK in the entire cohort is possibly due to influence from the adenocarcinoma cohort. In the Asian cohorts previously reported, approximately 50% patients were diagnosed with adenocarcinoma, which is a higher percentage than that found in our cohort. This fact suggests that the prognostic effect of pERK for NSCLC, reported in these studies, may be due to the influence of adenocarcinoma tumors in these cohorts. The most incident molecular alterations in lung adenocarcinoma, namely, KRAS mutations, EGFR mutations and ALK translocations, have been linked to MAPK activation [12–14]. Mutations in these three genes alone represent the driving alteration in approximately half of adenocarcinoma tumors [29], which is in accordance with the prognostic role of pERK observed in our adenocarcinoma cohort. Thus, our data suggest a potential central role for ERK in early-stage lung adenocarcinoma tumorigenesis and highlight a therapeutic potential for MEK inhibitors in these patients.

Additionally, we report a prognostic role for p53 nuclear expression in the lung squamous cell carcinoma patients from our cohort. It has been reported that p53 mutations confer stability to the protein and make it detectable by IHC staining [30-32]. The potential prognostic role of p53 immunostaining in NSCLC has been previously addressed in the literature, with controversial results. In multiple retrospective studies, no association was reported between high p53 expression and prognosis [33-36]. In these studies, adenocarcinoma, squamous cell carcinoma and other NSCLC histologies were jointly analyzed, which may explain that no prognostic potential could be reported for p53 immunostaining, consistent with our analysis for the entire cohort. In contrast to the previously cited studies, another study correlated p53 IHC and poorer survival in NSCLC [37]. This study evaluated p53 IHC not only in primary lung tumor samples but also in metastatic tumors, and most samples were obtained from advanced-stage tumors (III-IV). Furthermore, the different histologies of NSCLC were analyzed together, the most prevalent histology being adenocarcinoma. This study suggests a potential prognostic role for p53 immunostaining in advanced and metastatic NSCLC. In the present study, however, we aimed to identify early prognostic biomarkers. Therefore, we analyzed primary, resectable tumors, mainly early-stage tumors. We aimed to evaluate different histologies of NSCLC independently, due to their distinct molecular nature. These different characteristics of the cited study and the present study may explain the differences in the results from these studies. In accordance with our results, a study involving stage I lung squamous cell carcinoma patients reported that high p53 IHC correlates with lower overall survival [38]. Our results in a higher number of patients, are in line with this prognostic role for p53 IHC in lung squamous cell carcinoma and extend it to early-stage patients, and not only to stage I patients, with this subtype of NSCLC. Furthermore, we reported that high p53 immunostaining correlated with lower tumor differentiation when analyzing the entire NSCLC cohort; however, when we analyzed the adenocarcinoma and squamous cell carcinoma patient subsets independently, we observed that this correlation between p53 and tumor differentiation was only maintained in squamous cell carcinoma. p53 IHC staining has been previously related to poor differentiation in NSCLC [39]. Wild-type p53 has been involved in differentiation [40, 41] through the suppression of NANOG expression in mouse embryonic stem cells. Furthermore, mutant p53 has been shown to exert differentiation-blocking effects, affecting normal cellular maturation and generating highly proliferative lethal tumors [42] and to facilitate reprogramming efficiency of somatic cells [43-45]. These data suggest that p53 may have a role in tumor differentiation in squamous cell carcinoma tumors, which may explain its prognostic role that we report in this context. Regarding the potential therapeutic relevance of these findings, although limited therapies addressing p53 alterations have been approved for use in patients, several approaches to target p53 are being evaluated [46]. Our data suggest that selected squamous cell carcinoma patients may benefit from these therapies.

On the other hand, the determination of genome-wide mRNA expression in different NSCLC-patient cohorts has identified a number of prognostic multigene signatures stratifying early-stage NSCLC patients into different risk groups. In a recent large-scale meta-analysis comparing 42 published gene signatures in a large group of datasets, including 1927 NSCLC patients, the meta-estimated hazard ratios (95% CI) for predicted high risk groups was observed to be between 1.25 and 1.72 in the cases of adenocarcinoma-based signatures and between 1.25 and 1.41 for those signatures based on squamous cell carcinomas [47]. However, to date, only two multigene prognostic signatures have been commercialized and are currently under validation in prospective randomized controlled trials [48, 49]. These are myPlan® Lung Cancer (Myriad, Salt Lake City, UT) and Pervenio™ Lung RS platform (Life Technologies, West Sacrament, CA. Both signatures, focused on adenocarcinoma tumors, stratify patients into high- and low-risk groups with hazards ratios of approximately 1.5-2 [50, 51] and 2 [52, 53], respectively. However, we propose a simpler evaluation strategy (pERK in adenocarcinoma, and nuclear p53 in squamous cell carcinoma) for prognostic biomarkers using immunohistochemistry, a technique routinely performed in clinics and feasible in one FFPE sample sheet and thus requiring a very limited amount of tissue. Although validation in larger cohorts is required to confirm our results, our proposed prognostic marker for adenocarcinoma, pERK, shows an independent prognostic potential with a hazard ratio of approximately 4 in progression-free and overall survival. The result suggests that this single protein biomarker may predict effects more distinctly than either commercial gene signature. In addition, we propose p53 nuclear protein expression as a prognostic biomarker for early-stage squamous cell carcinoma tumors, a histologic subtype that may be excluded from these commercially available gene signature tests. This potential biomarker appears to predict a higher effect on prognosis, showing a hazard ratio of 2 for progression-free survival, above the 1.4 maximum value reported for the squamous cell lung carcinoma gene signatures aforementioned [47].

In addition, we assessed the potential of the expression of the proteins under study in predicting the response to adjuvant chemotherapy. We observed a clear trend suggesting that only patients with tumors exhibiting high nuclear pAKT or low pERK expression may benefit from this treatment, while this therapy may not be a good option for tumors with the opposite characteristics. In line with these results, high pAKT levels have been linked to better response to neoadjuvant chemotherapy in breast cancer patients [54], and low pERK expression has been correlated with longer survival of NSCLC patients receiving neoadjuvant chemotherapy [55]. However, other retrospective studies have shown contrasting results, suggesting that high-pERK- and low-pAKT-expressing NSCLC tumors are the tumors that respond to adjuvant chemotherapy [28]. Nevertheless, our analyses regarding the predictive value of these markers did not reach statistical significance, probably due to the low number of patients receiving adjuvant chemotherapy in our cohort. Due to this limitation, these results should be carefully interpreted, and further studies need to be conducted involving cohorts with a higher number of patients to evaluate the potential predictive role of these two markers for adjuvant chemotherapy efficacy.

In conclusion, we propose the immunohistochemical determination of the protein expression of pERK and nuclear p53 as potential prognostic biomarkers in resected adenocarcinoma and squamous cell lung carcinoma tumors, respectively. Determination of the expression of these potential biomarkers in the appropriate histologic context, through a technique demanding low sample quantity and used routinely in the clinic, may define the outcome of early-stage NSCLC patients and may offer a therapeutic opportunity to enhance survival of patients who undergo tumor surgical resection.

## MATERIALS AND METHODS

### Clinical specimens

The present study was performed in 248 early TNM stage (I-IIIA) subjects from the Virgen del Rocio Hospital (Seville, Spain), who had undergone surgical resection. Inclusion criteria were the following: (1) histologically confirmed diagnosis of early-stage NSCLC, (2) adequate clinical data recorded in medical charts, and (3) adequate tissue specimen available for immunohistochemistry (tissues were formalin-fixed and paraffin-embedded (FFPE) until further use). A written consent form for biobanking was obtained from all patients, and the study was approved by the Ethical Committee of the Virgen del Rocío Hospital.

### Immunohistochemistry

Tumoral area from the FFPE samples was identified by pathologists following hematoxylin-eosin staining. Tissue microarrays (TMAs) were constructed with punches of 1 mm diameter and 3 mm length obtained from the preselected tumoral area from every biopsy. Tissue processing was performed, while protecting samples from oxidation and maintaining the integrity of each sample during the process. De-paraffination and antigenic epitope recovery was performed using the PTLinK kit (Dako Glostrup, Denmark). Immune detection was performed with the pAKT (Ser473, #736E11, Cell Signaling), pERK (Thr202/Tyr204, #9101 CST), FLEX p53 (#GA616, Dako) and EGFR (#NCL-L-EGFR-384, Leica) antibodies. Scoring of IHC staining is based on the criteria followed in a previous study [56], as presented in Supplementary Table 1.

### Statistical analysis

Statistical analysis of clinical data and survival was performed with the SPSS statistical package (v19, IBM). The relationship between clinicopathological features and IHC data was analyzed using contingency tables, and p-value was obtained using the Chi-Square test. The magnitude of the effect is shown as odds ratio (OR) [95% confidence interval (CI)]. Kaplan-Meier overall survival (OS) and progression-free survival (PFS) curves were defined, and significant differences were calculated by a Log Rank univariate analysis. In addition, multivariate analysis was performed with the Cox proportional hazards method. In these analysis, OS and PFS were defined as the time from diagnosis to exitus and progression, respectively. P-values below 0.05 were considered as statistically significant.

### Availability of data and materials

The datasets generated and/or analyzed during the current study are available from the corresponding author upon reasonable request.

## SUPPLEMENTARY MATERIALS FIGURES AND TABLES


