# Supplementary material for: Histology-dependent prognostic role of pERK and p53 protein levels in early-stage non-small cell lung cancer
Source: Oncotarget. 2018 Apr 13;9(28):19945–60. doi: 10.18632/oncotarget.24977 (PMC5929438; doi:10.18632/oncotarget.24977)
Supplement: Supplementary file 1 [file oncotarget-09-19945-s001.pdf]

Histology-dependent prognostic role of pERK and p53 protein levels in early-stage non-small cell lung cancer

SUPPLEMENTARY MATERIALS

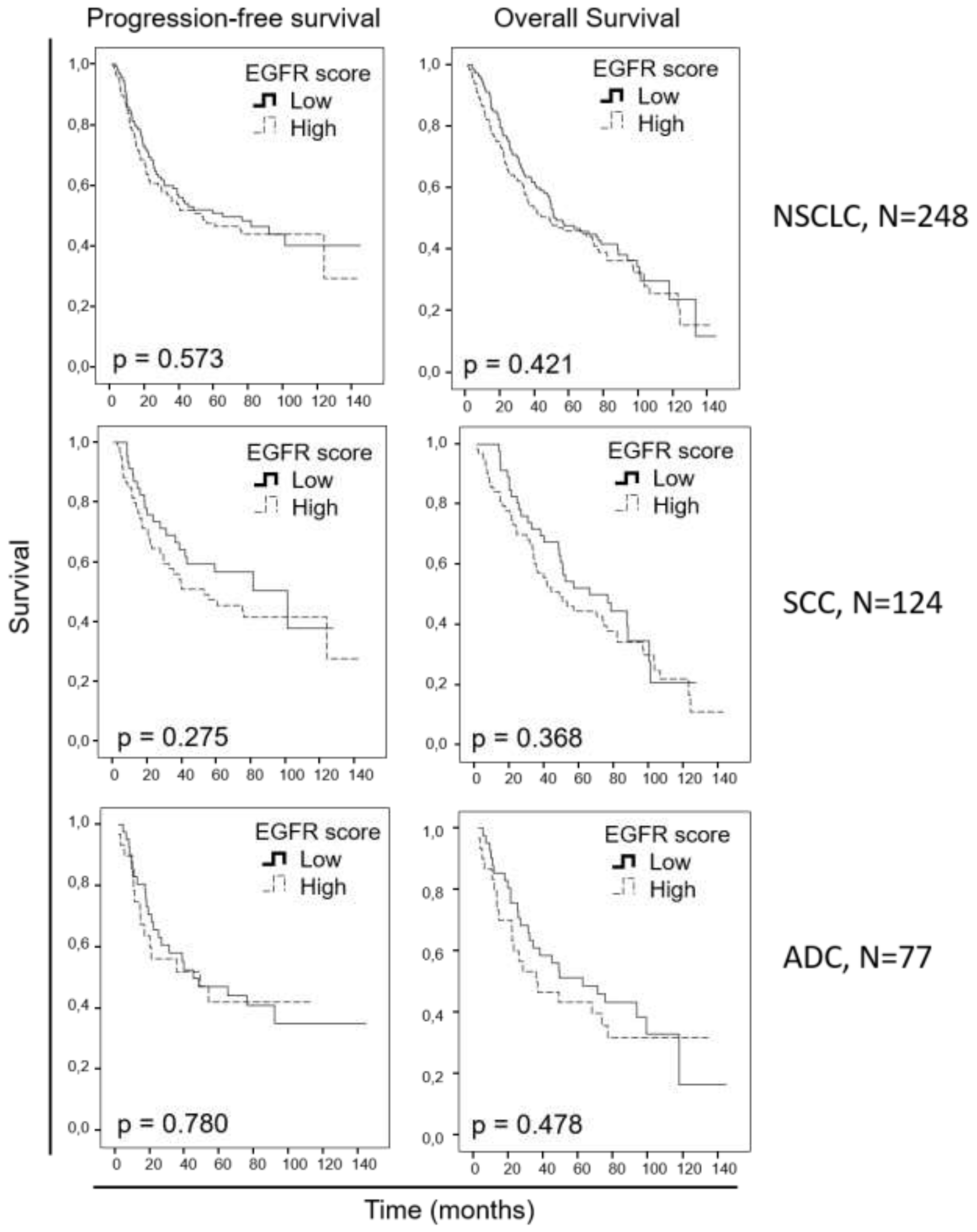

Supplementary Figure 1: Overall and progression-free Kaplan-Meier survival curves for the whole NSCLC patient cohort, and for the squamous cell carcinoma (SCC) and adenocarcinoma (ADC) patient subsets, attending to EGFR score, as assessed by IHC. Scores of 0 and 1 were considered as “low” and of 2 or 3 were considered as “high”.

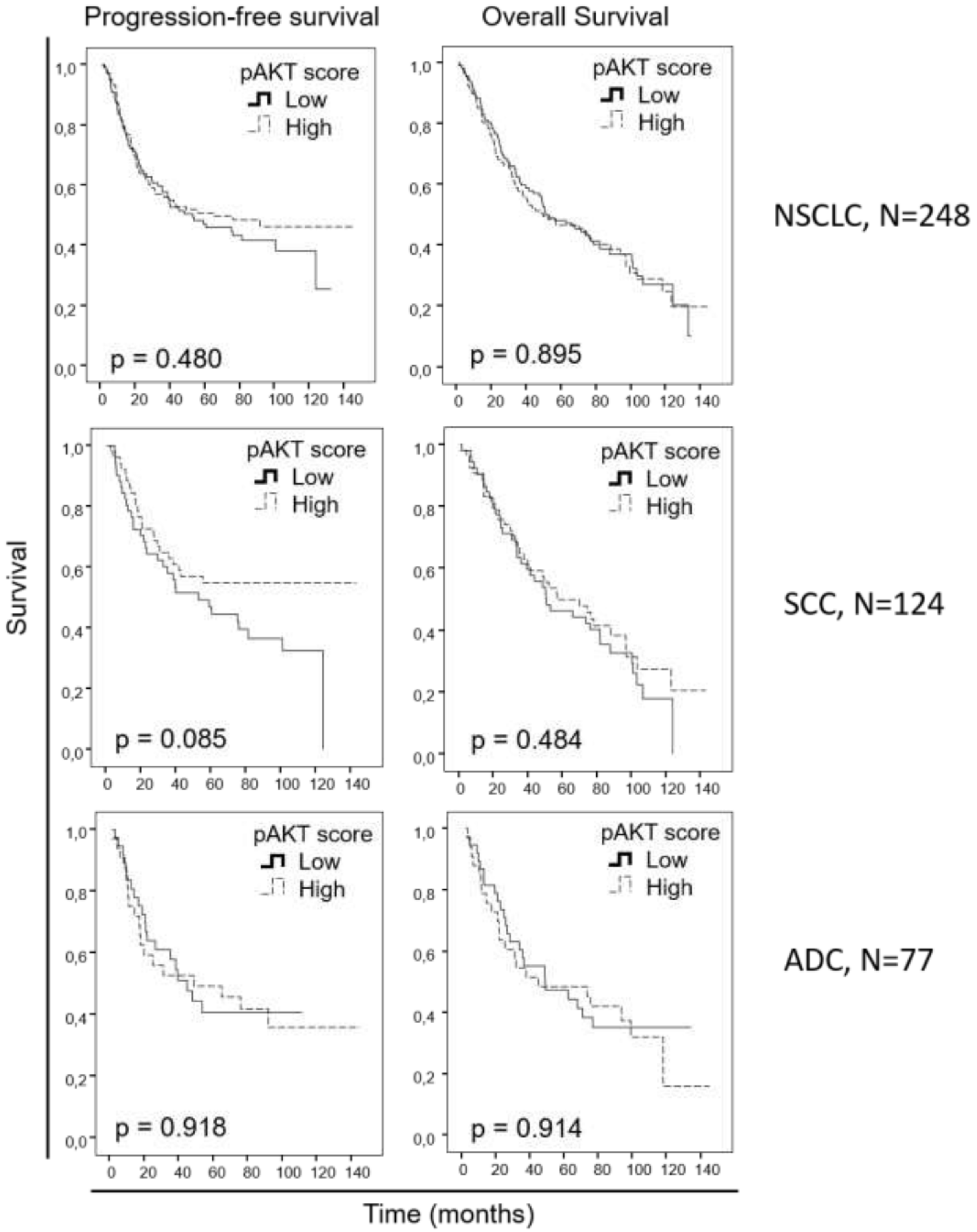

Supplementary Figure 2: Overall and progression-free Kaplan-Meier survival curves for the whole NSCLC patient cohort, and for the squamous cell carcinoma (SCC) and adenocarcinoma (ADC) patient subsets, attending to pAKT score, as assessed by IHC. Scores of 0 and 1 were considered as "low" and of 2 or 3 were considered as "high".

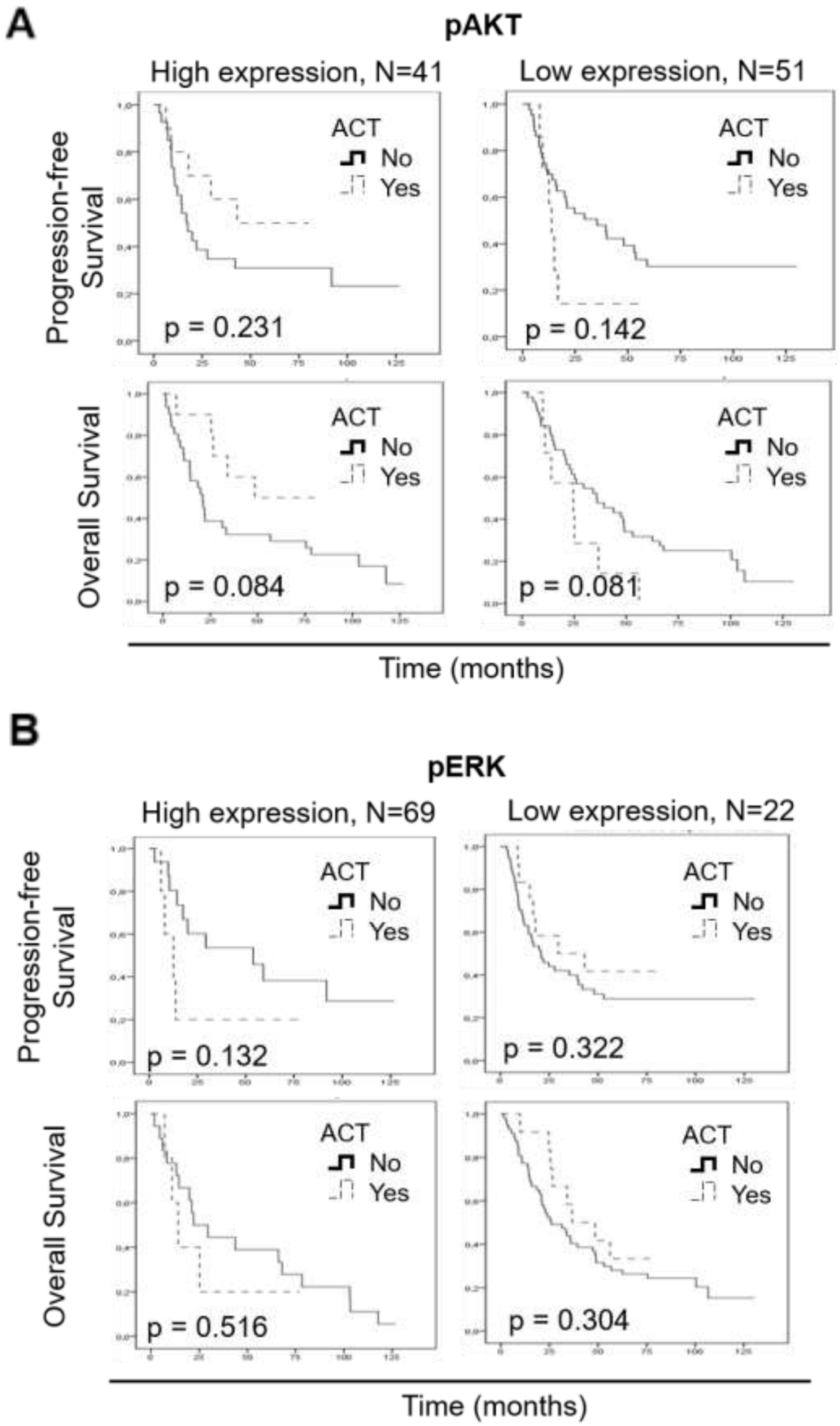

Supplementary Figure 3: Overall and progression-free Kaplan-Meier survival curves for the stage II-III NSCLC cohort subset divided by the protein expression levels of nuclear pAKT **(A)** or pERK **(B)**, attending to the administration or not of adjuvant chemotherapy. ACT = Adjuvant chemotherapy, High expression = high protein expression of the marker under study.

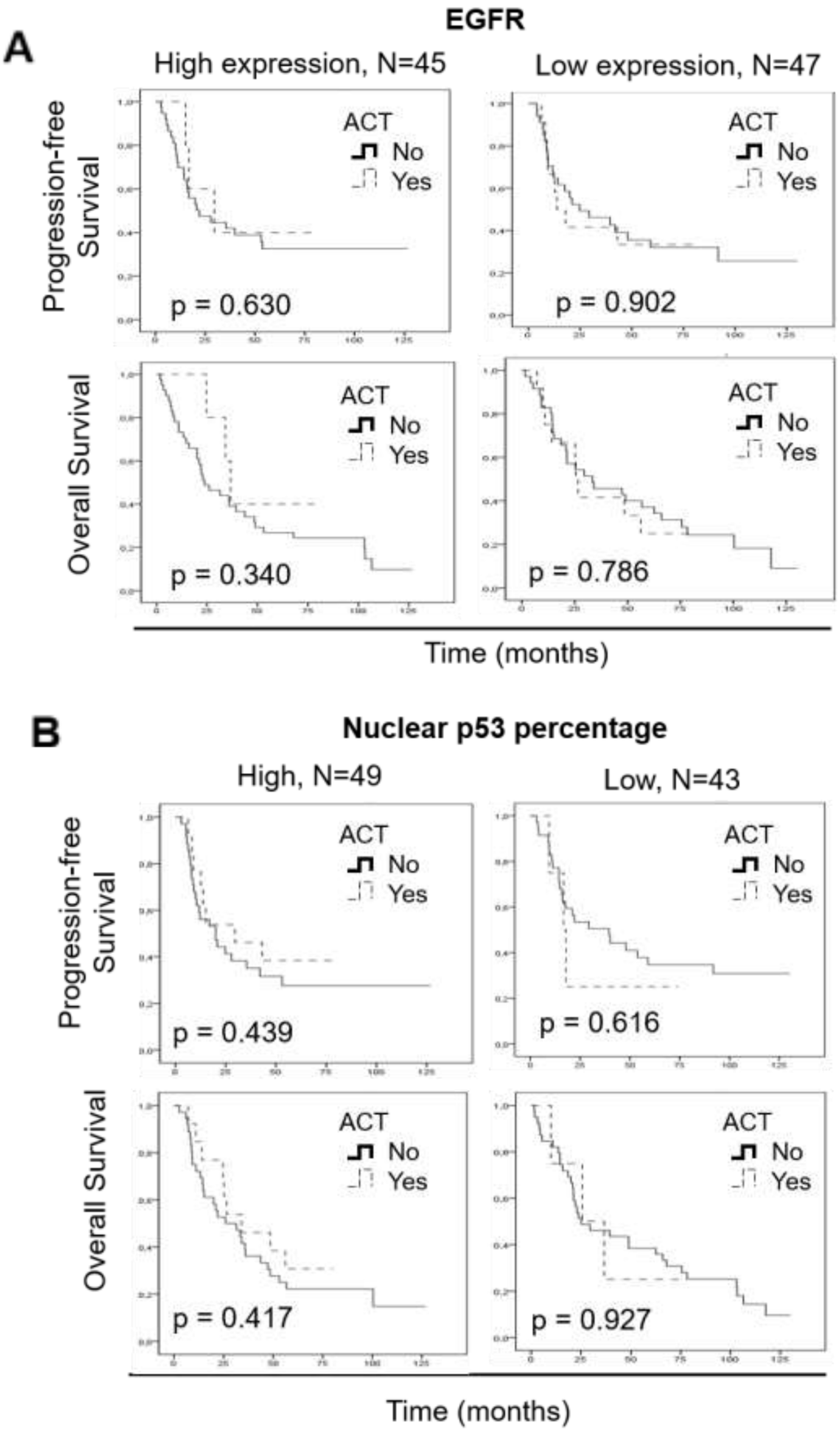

Supplementary Figure 4: Overall and progression-free Kaplan-Meier survival curves for the stage II-III NSCLC cohort subset divided by the protein expression levels of nuclear EGFR **(A)** or nuclear p53 percentage **(B)**, attending to the administration or not of adjuvant chemotherapy. ACT = Adjuvant chemotherapy.

Supplementary Table 1: Scoring criteria of IHC staining

Intensity	Percentage	Expression level
0	<10%	0
1	≥10%	1
2/3	<10%	2
2/3	≥10%	3
